# A Case Report of Takotsubo Cardiomyopathy With Myxedema Coma

**DOI:** 10.7759/cureus.32652

**Published:** 2022-12-18

**Authors:** Andrea C Marin, Sharon Hechter, Ankita Prasad, Ayesha Samad, Lee Manchio, Desai V Jiang, Arthur okere, Pramil Cheriyath

**Affiliations:** 1 Internal Medicine, Hackensack Meridian Health Ocean Medical Center, Brick, USA; 2 Cardiology, Hackensack Meridian Health Ocean Medical Center, Brick, USA

**Keywords:** apical balloning, angiography, stemi, hypthyroidism, myxedema coma

## Abstract

The exact pathogenesis of Takotsubo cardiomyopathy (TC) or broken heart syndrome is unclear. However, it is known to be a stress-induced cardiomyopathy. There are multiple causes of TC, and thyroid dysfunction is supposed to be one of the causes. We present a case of a 74-year-old female with a medical history of hypothyroidism who was admitted to the hospital with a myxedema coma and myocardial infarction. Her angiography had no evidence of plaque, thrombus, or spasm, and echocardiography showed apical ballooning, thus confirming the diagnosis of TC.

## Introduction

Takotsubo cardiomyopathy (TC), or broken heart syndrome, was first described in Japan by Dole et al. in 1991, and since then, there has been an increase in diagnosis. It is a stress-induced cardiomyopathy that causes chest pain and myocardial infarction (MI) in up to 10% of women with MI [1]. Most cases of TC are seen in postmenopausal women in their sixth decade. A left ventricular (LV) wall motion defect with LV dysfunction, new ST and T-wave abnormalities on the Electrocardiogram (ECG), an elevated troponin level, and no plaque, thrombus, or spasm on the coronary angiogram are seen. It is associated with intense emotional/physical stress, severe illness, or drug use which activates a catecholamine surge, causing cardiotoxicity and cardiac dysfunction. TC has been frequently associated with many endocrine disorders, including adrenal insufficiency, diabetes mellitus, autoimmune polyendocrine syndrome, and pheochromocytoma. Thyroid disorders, including hyperthyroidism, thyrotoxicosis, and hypothyroidism, are frequently seen in many patients with TC.

Myxedema coma is a life-threatening condition in patients with hypothyroidism. It occurs in severe illness, trauma, drugs, or noncompliance with thyroid medication. Patients usually present with altered mental status, hypothermia, hypotension, bradycardia, and myxedema. Laboratory studies show elevated thyroid-stimulating hormone (TSH), low T4, hyponatremia, and a low cortisol level. Treatment involves immediate levothyroxine, hydrocortisone, fluid resuscitation, rewarming, and ventilatory support if indicated. We present a case of TC with myxedema coma. This case is significant because most of the documented TCs with thyroid disorders are with hyperthyroidism, and there is a lack of literature on TC with hypothyroidism. Even after using Pubmed, Google Scholar, Embase, and Cochrane to search for cases of TC with myxedema, we could only find two documented cases of TC with hypothyroidism, reported by Salazar et al. [2] and Eric et al. [3], despite a population-based study using NIS data identifying more than 3000 cases [4] of hypothyroidism with TC.

## Case presentation

**Figure 1:**
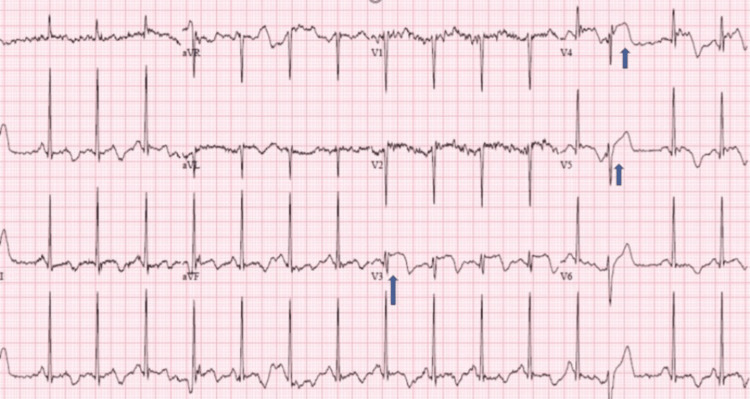
ECG at admission showing ST elevation in anterior leads V3, V4, V5 (blue arrows)

Our patient is a 74-year-old female brought into the emergency department after being found unconscious on the bathroom floor at home. At the presentation, she had altered mental status, was hypothermic (93 degrees Fahrenheit), and was hypertensive (blood pressure - 185/97mm Hg). Her heart rate was 90/minute, her respiration rate was 14/minute, and she maintained oxygen saturation at room temperature. Her skin was dry and rough. CNS examination was inconclusive. She had a past medical history of hypothyroidism and was on levothyroxine tablets (150 mcg/day). Due to her mental status, we could not get a record of medication compliance or review her other systems. Laboratory examination at admission showed leukocytosis (12.6x10^3^/mm^3^), serum sodium 163mmol/L, blood urea nitrogen/creatinine 149 mg/dL (6-24 mg/dL) /3.47 mg/dL (0.59 to 1.04 mg/dL) TSH 168 mIU/mL, free T4 0.33 ng/dL (0.7 to 1.9ng/dL), troponin of 12 (<0.04). Her ECG showed ST elevations in the anterior leads, V3 and V4 (Figure 1).

**Figure 2:**
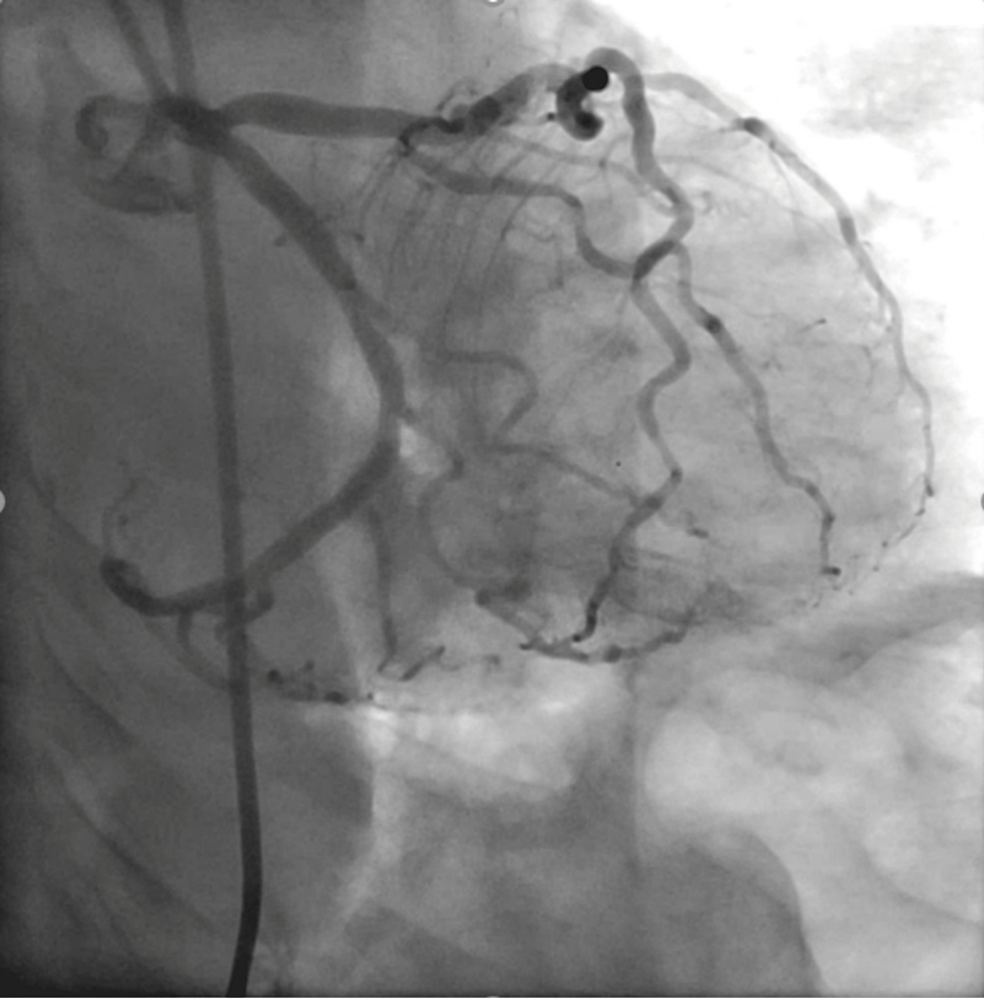
Normal coronary angiographic images from the patient

**Figure 3:**
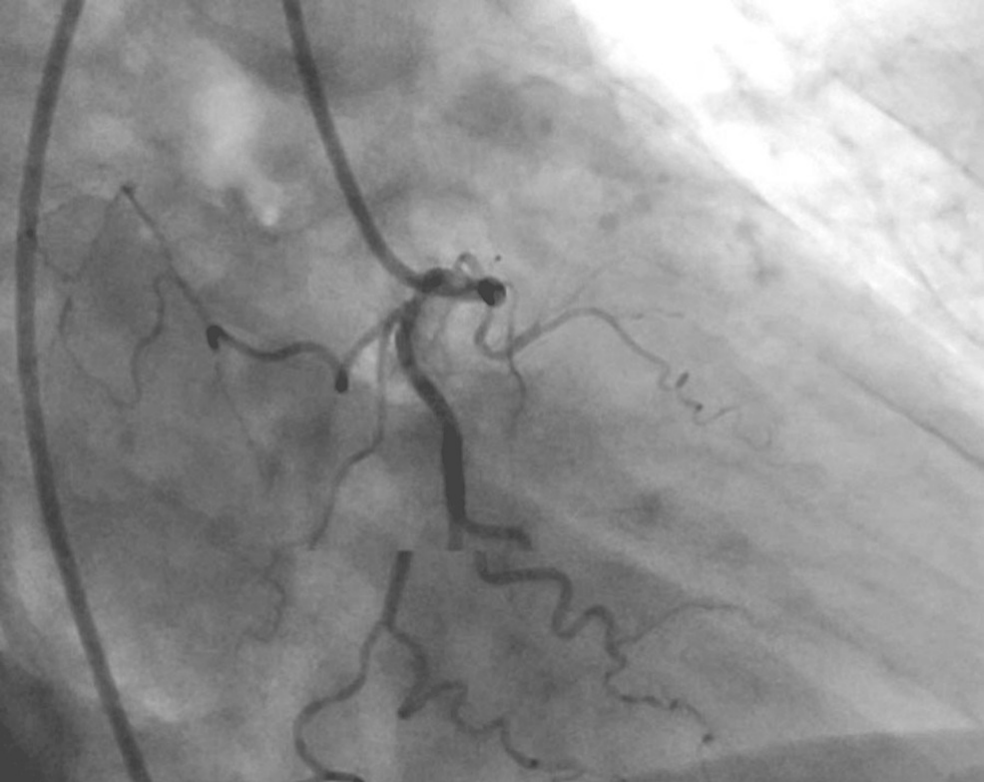
Normal coronary angiographic images

Thus, she had a myxedema coma with an ST-elevation MI. She underwent emergent cardiac catheterization, which showed no plaque, thrombus, spasm, or dissection in her coronary circulation (Figures 2, 3).

**Figure 4:**
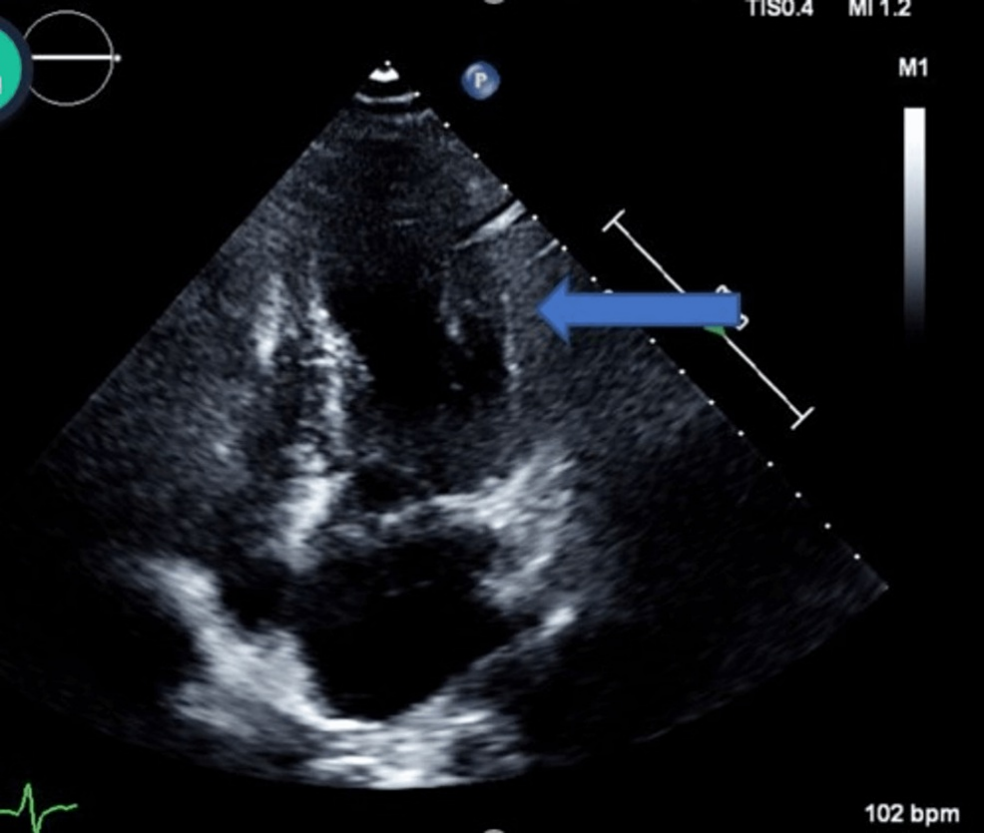
Transthoracic echocardiography showing apical balloning (blue arrow)

Transthoracic echocardiography showed apical ballooning with a hyperkinetic base and moderately reduced EF of 36% to 40%, confirming the diagnosis of TC (Figure 4).

**Figure 5:**
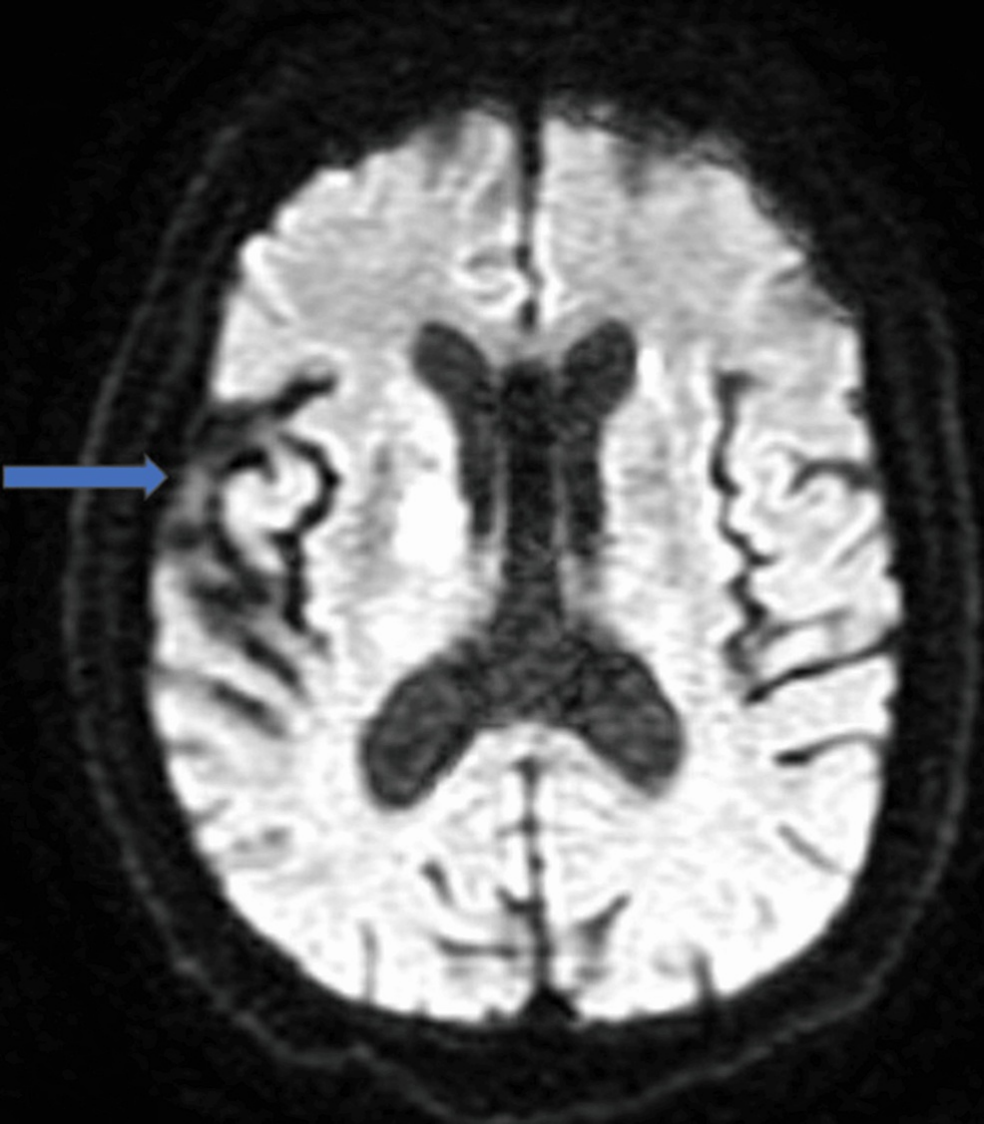
MRI brain showing infarct (blue arrow)

**Figure 6:**
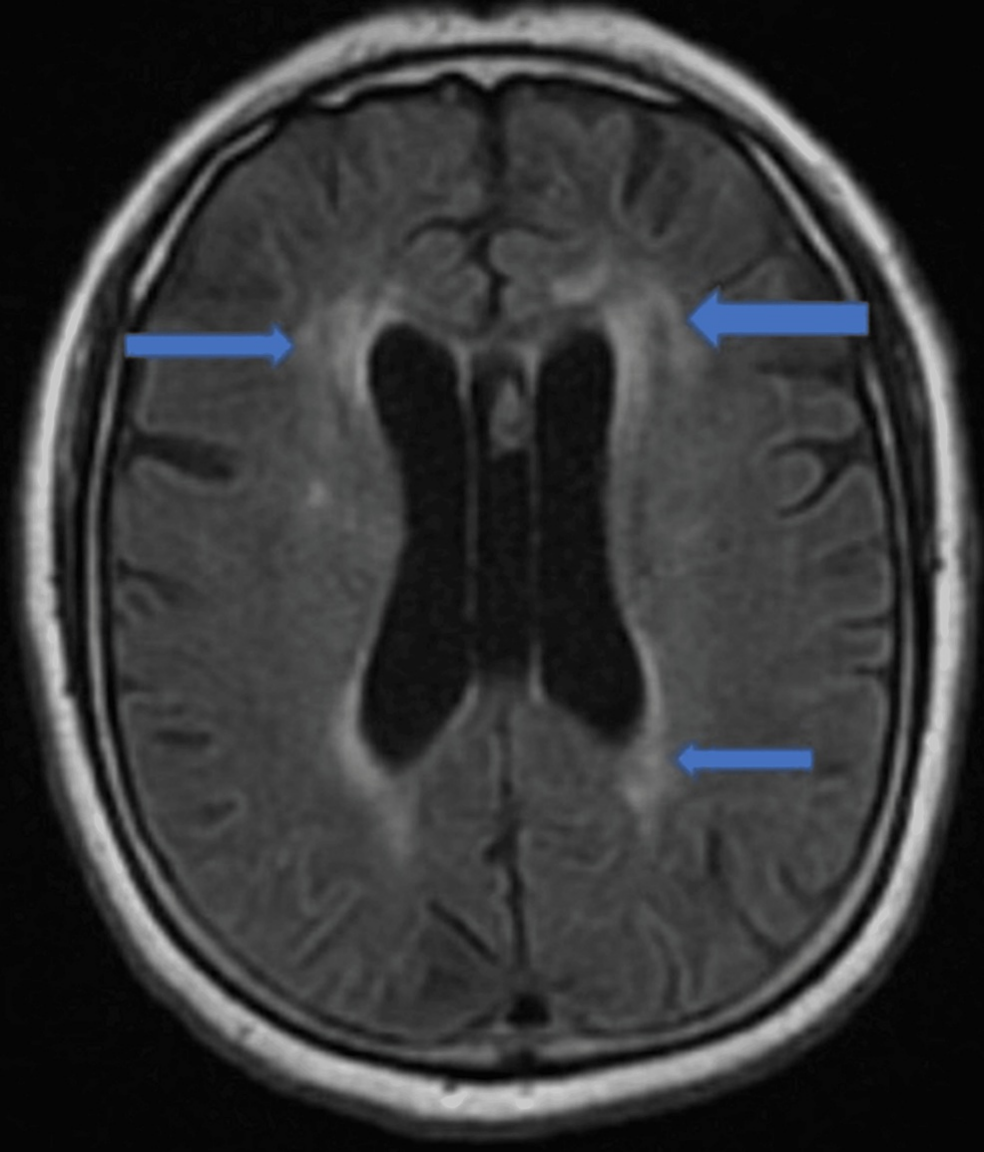
MRI brain showing bilaterally increased signal intensity (blue arrow)

She was managed conservatively with beta-blockers, angiotensin-converting enzyme inhibitors, and aspirin. Her computed tomography (CT) head was unremarkable. She was subsequently transferred to the intensive care unit, where she continued to be treated for her myxedema coma with intravenous levothyroxine and hydrocortisone. However, she remained confused, aphasic, and minimally responsive; therefore, we did a magnetic resonance imaging (MRI) of the brain without contrast, which showed two acute infarcts in the left caudate region and moderate white matter disease, raising the possibility of demyelinating disease. There was a bilaterally increased T2 signal intensity in basal ganglia (Figures 5, 6).

CT angiography of the head/neck showed intracranial stenosis in the left M2 segment. A transesophageal echocardiography was performed to rule out a cardioembolic source that showed an aortic plaque. No atrial septal defect, persistent foramen ovale, or any other cardiac sources for emboli were seen. Cardiologists recommended an implantable loop recorder (ILR) device to investigate possible atrial fibrillation. A venous ultrasound was performed, which revealed a left posterior tibial deep vein thrombosis; she was placed on apixaban and discharged in a stable state with advice to follow up.

## Discussion

TC is diagnosed by clinical and electrocardiographic features of myocardial ischemia (including ST segment depression or elevation and T-wave inversions), the elevation of cardiac enzymes, apical systolic ballooning with preserved or increased contractility at the base, and no evidence of obstructive coronary artery disease on angiography. Several TC variants have been described. In the typical or apical variation, a hyperkinetic LV base with focal apical akinesis causes apical ballooning and a reduced ejection fraction [5]. Another version is the inverted or basal pattern (circumferential basal). The mid-LV variety (circumferential midventricular hypokinesis and basal and apical hypercontractility), the biventricular apical and right ventricular pattern, and the biventricular apical and right ventricular pattern have all been described [5]. Multicentric studies have shown a recurrence rate of 4% and up to 20% in cases of variable TC [6].

Although the exact pathophysiology of TC is unknown, it has been linked to hormonal dysfunction. The primary trigger appears to be excessive neurogenic sympathetic activation under physical or emotional stress that causes neurogenic myocardial stunning with aberrant glucose and fatty acid metabolism, which is mediated by catecholamine-induced enzymatic and surface receptor alterations [2]. Transient microvascular dysfunction has been documented in TCM patients using adenosine and myocardial contrast echocardiography [2,7]. Pituitary, thyroid, adrenal, and estrogen metabolic disorders are associated with TC cases. TC is more common in women with hypothyroidism. In a retrospective study, Aggarwal et al. found an association between TC with thyroid disorders. They reported 35% of the patients to be hypothyroid, with the majority of them being on thyroid replacement and 11.36% being in a hyperthyroid state [8]. Myxedema coma has multiple effects on the body, causing coronary artery vasospasm, decreased heart contractility, decreased cardiac output, and increased systemic vascular resistance. A mechanism where myxedema coma could have potentially induced TC in this patient is coronary artery vasospasm and a decrease in ventricular contraction, cardiac dilation, and cardiac atrophy. Hypothyroidism leads to cardiovascular mortality in many ways, and even subclinical hypothyroidism is independently related to the prevalence of aortic atherosclerosis and MI in older women, according to the Rotterdam research, a large epidemiological cohort from the Netherlands [9]. Coronary flow reserve is markedly diminished in hypothyroidism, regardless of lipid profile [10]. Studies have reported alterations of the autonomic nervous system in patients with hypothyroidism, resulting in an imbalance of vagal and sympathetic modulation [11,12]. All these pathological processes contribute to the development of Takotsubo.

## Conclusions

Myxedema coma is a severe form of hypothyroidism that affects the human body in multiple ways. It causes immense stress to the body. Although the exact pathophysiology of TC is unclear, it is known to occur with high physical and emotional stress. In the most commonly seen variants, there is an apical ballooning with a hyperkinetic base along with no spasm plaque or thrombus seen on angiography. There have been multiple endocrine dysregulation syndromes that have been known to cause TC. Hypothyroidism has been associated with TC.
