# Correction: A Case Report of Takotsubo Cardiomyopathy With Myxedema Coma

**DOI:** 10.7759/cureus.c105

**Published:** 2023-03-03

**Authors:** Andrea C Marin, Sharon Hechter, Ankita Prasad, Ayesha Samad, Lee Manchio, Desai V Jiang, Arthur okere, Pramil Cheriyath

**Affiliations:** 1 Internal Medicine, Hackensack Meridian Health Ocean Medical Center, Brick, USA; 2 Cardiology, Hackensack Meridian Health Ocean Medical Center, Brick, USA

This article has been corrected at the request of the authors to edit the name of the heart condition in the title from “Takotsubo’s Cardiomyopathy to “Takotsubo Cardiomyopathy”.

The authors deeply regret that this error was not identified and addressed prior to publication.

 

